# Pet Birds as Potential Reservoirs of Antimicrobial-Resistant Bacteria in Digestive and Respiratory Infections

**DOI:** 10.3390/antibiotics15050487

**Published:** 2026-05-12

**Authors:** Smaranda Crăciun, Maria Carmen Turcu, Cristiana Ştefania Novac, Nicodim Iosif Fiţ, Cosmina Maria Bouari, Sorin Răpuntean, Mălina Lorena Mihu, George Cosmin Nadăş

**Affiliations:** 1 Department of Microbiology, Immunology and Epidemiology, Faculty of Veterinary Medicine, University of Agricultural Sciences and Veterinary Medicine, 400372 Cluj-Napoca, Romania; cristiana.novac@usamvcluj.ro (C.Ş.N.); nfit@usamvcluj.ro (N.I.F.); cosmina.bouari@usamvcluj.ro (C.M.B.); sorin.rapuntean@usamvcluj.ro (S.R.); malina.mihu@usamvcluj.ro (M.L.M.); gnadas@usamvcluj.ro (G.C.N.); 2 New Companion Animals Veterinary Clinic, Faculty of Veterinary Medicine, University of Agricultural Sciences and Veterinary Medicine Cluj-Napoca, 400372 Cluj-Napoca, Romania; maria-carmen.turcu@usamvcluj.ro

**Keywords:** pet birds, antimicrobial resistance, multidrug resistance, bacterial isolates, zoonotic pathogens

## Abstract

Background/Objectives: Pet birds are increasingly recognized as potential reservoirs of zoonotic and antimicrobial-resistant bacteria, raising concerns within the One Health framework. However, data on bacterial diversity and resistance profiles in clinically affected ornamental birds remain limited. Methods: This study, conducted over three years (November 2022–March 2026), included 198 pet birds presenting with digestive and respiratory disorders. From these birds, clinical samples were analyzed bacteriologically; resulting isolates were identified by MALDI-TOF mass spectrometry, and antimicrobial susceptibility assessed using the Kirby-Bauer disk diffusion method according to EUCAST and CLSI guidelines. Results: Bacterial growth was detected in 87.9% of cases, yielding 249 distinct isolates. Gram-positive cocci predominated (62.3%), led by *Staphylococcus* spp. (33.3%) and *Enterococcus* spp. (9.6%), while *Escherichia coli* (9.2%) was the primary Gram-negative species. At the genus level, *Staphylococcus* spp. demonstrated high susceptibility to amikacin (88.5%) but significant resistance to gentamicin (75.6%) and oxytetracycline (63.6%). In contrast, *Escherichia* spp. isolates were largely resistant, showing only 50% susceptibility to enrofloxacin and 40% to doxycycline, with resistance to tylosin reaching 90%. Overall, 57% of isolates were multidrug-resistant, with *Staphylococcus* spp. contributing most to this burden. Conclusions: These findings characterize clinically ill pet birds as significant carriers of multidrug-resistant bacteria, highlighting the need for routine diagnostics and improved antimicrobial stewardship in avian medicine.

## 1. Introduction

The global landscape of companion animals has undergone a significant transformation in recent years, expanding beyond traditional domestic species such as dogs, cats, and horses to include a wide range of non-traditional companion animals, commonly referred to as exotic pets [1,2]. Among these, ornamental birds, defined as species kept for purposes other than consumption, including budgerigars, cockatiels, larger parrots, finches, and canaries, have emerged as the third most common type of pet worldwide [3,4,5]. This diverse group is primarily composed of the orders *Psittaciformes*, which includes parrots, parakeets, and lovebirds, and *Passeriformes*, commonly known as songbirds [6,7]. Additionally, species belonging to the orders *Columbiformes*, such as pigeons, and *Galliformes* are increasingly maintained for ornamental purposes [7,8]. The growing popularity of these birds is largely driven by their capacity to form strong emotional bonds with their owners and to adapt to modern lifestyles, particularly as households increasingly favor smaller companion animals due to limited time and space [6,9]. However, this increasing human–animal proximity also creates conditions that facilitate the bidirectional transmission of bacteria and antimicrobial resistance [1,10,11]. Although both exotic and traditional pets are recognized as contributors to zoonotic disease transmission, awareness among owners regarding the risks associated with non-traditional companion animals remains limited [12].

The presence of diverse microorganisms within the avian microbiota represents a significant zoonotic risk, as pet birds can serve as potential and often overlooked reservoirs of enteric and systemic pathogens of considerable economic and public health importance [3]. In this context, it is critical to distinguish between normal microbiota or asymptomatic colonization and true clinical infection, where the isolated agent is the direct cause of the observed pathology. Numerous bacterial species are implicated in primary and secondary infections in companion birds, with *Escherichia coli*, *Klebsiella* spp., and *Pseudomonas aeruginosa* among the most common etiological agents associated with respiratory, gastrointestinal, and systemic disease [8,13]. Other Gram-negative bacteria, including *Bordetella* spp., *Salmonella* spp., *Proteus* spp., *Serratia* spp., *Yersinia* spp., and *Pasteurella* spp., are also frequently identified [13]. Among Gram-positive organisms, *Staphylococcus aureus* is the most prevalent pathogen, commonly associated with infections of the skin, upper respiratory tract, and gastrointestinal system, while *Enterococcus* spp. are occasionally linked to tracheitis in canaries and respiratory or osteoarticular infections in psittacine species [6,13]. Although *Escherichia coli* is a normal component of the avian intestinal microbiota, it remains a major cause of colibacillosis and septicemia and may act as a reservoir of virulent and antimicrobial-resistant strains with zoonotic potential [3,8]. Additionally, *Klebsiella pneumoniae* and methicillin-resistant *Staphylococcus aureus* (MRSA) have been isolated from birds, posing recognized risks to human contacts [14,15]. Given the close interaction between pet birds and their owners, the transmission of zoonotic and antimicrobial-resistant bacteria is plausible, particularly among vulnerable populations such as children, elderly individuals, pregnant women, and immunocompromised persons [11,16]. Nevertheless, zoonotic infections originating from pet birds remain comparatively under-investigated [3,7].

In avian clinical practice, antibiotics are commonly used to treat bacterial infections, and due to the relatively small size of companion birds, treatment costs are often less restrictive, allowing clinicians to select from a wide range of both veterinary- and human-labeled antimicrobial agents [13,17]. The selection of an appropriate antibiotic requires careful consideration of the etiological agent, the site and severity of infection, antimicrobial susceptibility, pharmacokinetic properties, and potential drug toxicity [13,17]. However, it is essential that antimicrobial therapy is initiated only when clearly indicated, as birds are often difficult to medicate and may exhibit nonspecific clinical signs. Furthermore, the mere isolation of a microorganism does not necessarily justify treatment, since low numbers of potentially pathogenic bacteria are frequently present in the choana and cloaca of clinically healthy birds [13]. Distinguishing these opportunistic isolates from environmental contaminants or normal flora is essential for accurate diagnosis.

Antimicrobial stewardship in exotic avian medicine is further complicated by the limited availability of species-specific guidelines and the frequent reliance on empirical prescribing [18,19]. One of the most commonly used antimicrobials in ornamental birds is enrofloxacin, a fluoroquinolone licensed for use in exotic pet medicine [5]. However, fluoroquinolones are classified by the World Health Organization as “highest priority critically important antimicrobials” for human medicine, and their use in food-producing animals, including poultry, is restricted or banned in several regions due to concerns regarding resistance development [5,20].

There is growing evidence that antimicrobial resistance (AMR) represents a major global public health threat, as recognized by the World Health Organization [12]. This concern is further amplified by the fact that AMR is currently ranked among the top ten global health challenges [10,21,22]. Although resistance can arise as a natural evolutionary process, it is significantly accelerated by the inappropriate use of antimicrobials, including non-prescription access, empirical therapy, and owner-driven misuse [1,23,24]. While companion animals are increasingly recognized as reservoirs of zoonotic bacteria, their role as carriers of multidrug-resistant (MDR) pathogens remains insufficiently characterized [12]. In particular, pathogens such as methicillin-resistant *Staphylococcus pseudintermedius* (MRSP), methicillin-resistant *Staphylococcus aureus* (MRSA), and multidrug-resistant Gram-negative bacteria such as *Pseudomonas aeruginosa* (classified as a critical priority pathogen) represent significant clinical and public health concerns [10,25]. In the context of exotic animal medicine, these challenges are further exacerbated by the absence of dedicated antimicrobial stewardship frameworks and standardized therapeutic guidelines [18,19].

Because antimicrobial-resistant bacteria can readily cross the boundaries between animals, humans, and the environment, the implementation of a One Health approach is essential, integrating infection prevention strategies and regulatory measures [10,21]. Without further investigation into the role of ornamental birds in the dissemination of resistant bacteria, these animals may continue to act as significant and under-recognized reservoirs of pathogens associated with treatment failure in both human and veterinary medicine [3,9,26].

Despite the growing recognition of pet birds as potential reservoirs of zoonotic and antimicrobial-resistant bacteria, data on the diversity of bacterial pathogens and their resistance profiles in clinically affected companion birds remain limited, particularly in Eastern Europe. Moreover, the relationship between clinical presentation and antimicrobial resistance patterns in these species is still poorly characterized. To our knowledge, this is one of the few studies in Eastern Europe to integrate clinical presentation, MALDI-TOF-based identification, and antimicrobial resistance profiling in pet birds within a One Health framework. Therefore, the present study aimed to test the hypothesis that clinically ill ornamental birds harbor significant frequencies of multidrug-resistant pathogens. Specifically, we aimed to investigate the bacterial etiology associated with digestive and respiratory disorders and to quantify the prevalence of multidrug resistance among the isolated strains.

## 2. Results

### 2.1. Clinical Presentation

The study population comprised a total of 198 birds, presenting with a variety of clinical conditions. In terms of gender distribution, a higher proportion of males was observed (59%, *n* = 117) compared to females (41%, *n* = 81). Regarding age, the majority of the avian patients were classified as adults over one year of age (69.7%, *n* = 138), while birds aged one year or younger accounted for 30.3% (*n* = 60) of the total. The clinical presentation was divided between scheduled appointments (68.7%, *n* = 136) and emergency cases (31.3%, *n* = 62).

The taxonomic representation was diverse, with budgerigars being the most prevalent group (54%, *n* = 107). Other species included parrots (18.7%, *n* = 37), cockatiels (17.2%, *n* = 34), and agapornis (6.1%, *n* = 12). Less frequently represented species were chickens (2%, *n* = 4) and pigeons (1.5%, *n* = 3), while a single canary was recorded (0.5%, *n* = 1). Regarding previous medical intervention, most patients (78.3%, *n* = 155) had not received antimicrobial therapy prior to the microbiological examination, while 21.7% (*n* = 43) had undergone antibiotic treatment before sampling.

The clinical focus of this study was centered on the two most prevalent health concerns in the avian population: digestive and respiratory disorders. Among the total cases analyzed, digestive pathologies remained the dominant clinical presentation, accounting for 62.1% (*n* = 123) of the birds. Respiratory affections constituted the remaining 37.9% (*n* = 75) of the study group. In terms of temporal distribution, the influx of cases remained relatively consistent throughout the year, with a slight peak observed during the warmer months. The summer season accounted for the highest proportion of clinical presentations at 27.3% (*n* = 54), followed closely by winter with 26.3% (*n* = 52). The remaining cases were distributed between autumn, representing 23.7% (*n* = 47), and spring, which accounted for 22.7% (*n* = 45) of the total admissions.

Overall, the study population was predominantly composed of adult male birds, most commonly budgerigars. Table 1 summarizes all of the study population’s data.

To further detail the clinical characteristics of the study population, the relationship between prior antimicrobial intervention and pathology was analyzed. Within the cohort of birds that had received antibiotic therapy prior to microbiological sampling (21.7%, *n* = 43), digestive disorders were the primary indication for treatment, accounting for 58.1% (*n* = 25) of the medicated group. Respiratory infections followed, representing 41.9 (*n* = 18) of the birds treated before the exam. These data suggest that approximately 21.7% of the avian patients presented with a history of recent antimicrobial exposure. While specific details regarding the class, dosage, and duration of these treatments were not available, this prior exposure should be considered as a potential confounding factor when interpreting the subsequent microbiological isolation rates and resistance profiles. The distribution of pathologies across demographics (Table 2) revealed highly consistent patterns. Among the 123 birds presenting with digestive signs, the majority were males (59.3%, *n* = 73) and adults over one year of age (69.9%, *n* = 86). A nearly identical trend was observed in the 75 respiratory cases, where males constituted 58.7% (*n* = 44) of the group and adults represented 69.3% (*n* = 52).

To determine if significant associations exist between the clinical pathologies and the demographic variables (sex, age, and season), a Chi-square (χ^2^) test of independence was performed. The analysis revealed no significant association between the gender of the birds and the type of pathology presented (χ^2^ < 0.001, *p* = 1.00). The Cramer’s V value of 0.00 indicates a negligible effect size, confirming that males and females are similarly predisposed to both digestive and respiratory conditions. Similarly, no statistically significant association was identified between age group (young vs. adult) and clinical pathology (χ^2^ < 0.001, *p* = 1.00; Cramer’s V = 0.00).

Regarding temporal factors, the time of year did not significantly influence the occurrence of specific pathologies (χ^2^ = 0.0275, df = 3, *p* = 0.9988). The Cramer’s V of 0.0118 confirms that seasonal variations, while affecting the total number of admissions (with slight peaks in summer and winter), do not specifically favor one type of infection over the other.

In conclusion, while the descriptive data showed a higher volume of digestive cases and a general male/adult predominance in the patient population, statistical analysis detected no significant associations between these demographic patterns and the specific pathology. Within the limits of this study cohort, sufficient statistical evidence was not found to suggest a biological or environmental link related to sex, age, or season for either digestive or respiratory presentations. Exploratory analysis using Odds Ratios (OR) revealed no significant demographic associations for either pathology. The likelihood of presenting with respiratory versus digestive disorders was equivalent across genders (OR = 1.02; 95% CI: 0.56–1.86) and age groups (OR = 1.03; 95% CI: 0.55–1.92). Additionally, prior antimicrobial exposure did not differ significantly between respiratory and digestive cases (OR = 1.18; 95% CI: 0.59–2.36). This exploratory comparison suggests that within this cohort, demographic factors did not specifically favor the occurrence of one pathology over the other.

### 2.2. Bacterial Identification

Of the 198 avian samples analyzed, 174 (87.9%) yielded positive bacterial growth, resulting in the identification of 249 isolates across 26 distinct genera via MALDI-TOF MS. Gram-positive cocci (*Dermacoccus*, *Enterococcus*, *Kocuria*, *Lactococcus*, *Micrococcus*, *Rothia*, *Staphylococcus*, *Streptococcus*) constituted the most prevalent group at 62.3% (*n* = 155) of the isolates, followed by the *Enterobacteriaceae* family (*Enterobacter*, *Escherichia*, *Klebsiella*, *Kosakonia*, *Leclercia*, *Mixta*, *Proteus*, *Raoultella*, *Serratia*) at 20.9% (*n* = 52), while Gram-positive bacilli (*Bacillus*, *Corynebacterium*, *Lactobacillus*, *Microbacterium*) represented 10% (*n* = 25) of the isolates. The remaining 6.8% (*n* = 17) of the isolates consisted of non-*Enterobacteriaceae* (*Acinetobacter*, *Aeromonas*, *Gallibacterium*, *Neisseria*, *Pseudomonas*). A total of 249 isolates were obtained, with 180 (72.3%) being Gram-positive and 69 (27.7%) Gram-negative, consisting of 74 species. The distribution of the isolated bacterial strains across the two pathologies is presented in Table 3. While all sampled birds presented with clinical signs, the isolated species were categorized based on clinical plausibility. The majority of cases involved a single bacterial isolate, though polybacterial infections were significantly represented. Specifically, 60.9% (*n* = 106) of the birds exhibited infection with a single pathogen. Coinfection with two distinct bacterial strains was observed in 34.5% (*n* = 60) of the cases, while 4.6% (*n* = 8) of the patients presented with a more complex clinical picture involving three distinct bacterial pathogens, suggesting the involvement of both primary pathogens and opportunistic commensal flora.

**Figure 1:**
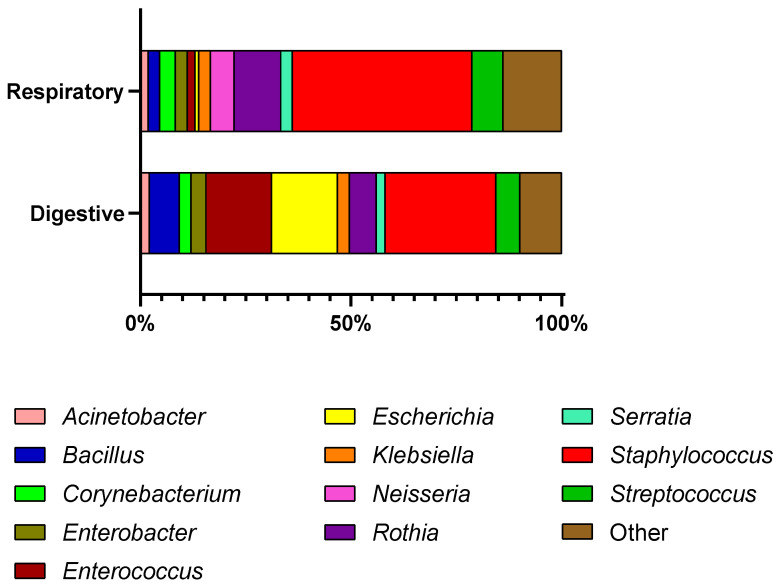
Distribution of bacterial genera according to sample origin in pet birds.

Among the 249 bacterial isolates, the most frequently recovered genus was *Staphylococcus*, accounting for 33.3% (*n* = 83), with *Staphylococcus haemolyticus* identified as the most prevalent species within the genus (31.3%, *n* = 26). This was followed by *Enterococcus* at 9.6% (*n* = 24), predominantly represented by *Enterococcus faecalis* (75.0%, *n* = 18). The genus *Escherichia* accounted for 9.2% (*n* = 23) of the total isolates, all of which were identified as *Escherichia coli* (100%, *n* = 23). Furthermore, *Rothia* was identified in 8.4% (*n* = 21) of cases, with *Rothia nasimurium* being the most common species (57.1%, *n* = 12), while *Streptococcus* was isolated in 6.4% (*n* = 16) of instances, led by *Streptococcus salivarius* (37.5%, *n* = 6). Collectively, these five genera represent 67.1% of the total microbial recovery, underscoring their primary role in the clinical landscape of the studied population, whereas the remaining comprised various rare or opportunistic species of uncertain clinical significance. All isolated bacterial species are listed in Table 3, along with the distribution of bacterial genera, which is illustrated in Figure 1.

### 2.3. Antibiotic Susceptibility Testing

The interpretation of antimicrobial susceptibility results followed a hierarchical approach across four taxonomic categories: Gram-positive cocci, Gram-positive bacilli, *Enterobacteriaceae*, and non-*Enterobacteriaceae*. EUCAST (2026) [27] served as the primary interpretive standard for pathogens of high zoonotic significance to ensure alignment with international One Health surveillance frameworks. For antimicrobial agents or bacterial species not comprehensively covered by EUCAST, specifically veterinary-specific molecules, CLSI VET01S (6th ed.) breakpoints were utilized. In cases where specific breakpoints for avian isolates were unavailable, criteria were extrapolated from the most biologically and pharmacologically relevant standards, such as feline/canine criteria for *Staphylococcus* and *Enterobacterales*, or poultry standards for *Escherichia coli*, as per CLSI recommendations. Discrepancies between the two standards were resolved by prioritizing EUCAST for global consistency, while CLSI VET standards were preferred when they provided a more accurate pharmacological representation for veterinary-specific applications. For bacterial species or agents lacking defined interpretive criteria in either standard, inhibition zone diameters were reported as continuous variables, and any tentative interpretations were considered strictly exploratory and treated with particular caution. Taxon-specific analysis of the 249 avian isolates revealed significant acquired resistance across all bacterial groups. To ensure analytical accuracy, known intrinsic resistances were excluded from the susceptibility calculations and MDR characterization.

**Figure 2:**
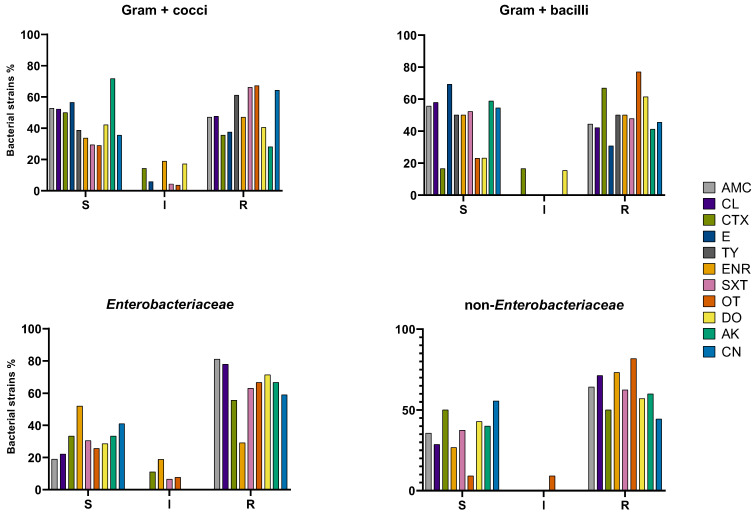
Antimicrobial-resistant bacterial strains (%) from each group (G + cocci, G + bacilli, Enterobacteriaceae, non-Enterobacteriaceae). R-resistant; S-susceptible; I-intermediate; AMC-amoxicillin/clavulanic acid; LEX-cephalexin; CTX-cefotaxime; ERY-erythromycin; TYL-tylosin; ENR-enrofloxacin; SXT-trimethoprim/sulphamethoxazole; OXY-oxytetracycline; DOX-doxycycline; AMK-amikacin; GEN-gentamicin.

The antimicrobial susceptibility analysis of the 249 avian isolates revealed significant resistance across all bacterial groups, with Gram-negative bacteria exhibiting the most critical profiles. Within the *Enterobacteriaceae*, resistance was most pronounced for cephalexin (71.4%) and amoxicillin/clavulanic acid (81%), while enrofloxacin showed the highest susceptibility (52%). A similar trend was observed in non-*Enterobacteriaceae*, where oxytetracycline resistance reached 81.8%, followed by enrofloxacin (73.3%). Among the Gram-positive cocci, resistance to oxytetracycline (67.3%) and tylosin (61.2%) was prevalent, whereas amikacin remained the most effective agent with 71.8% susceptibility. In the Gram-positive bacilli group, oxytetracycline resistance was high (77%), contrasted by strong susceptibility to erythromycin (69.2%). For a better visualization and data interpretation, the results are graphically presented in Figure 2.

**Figure 3:**
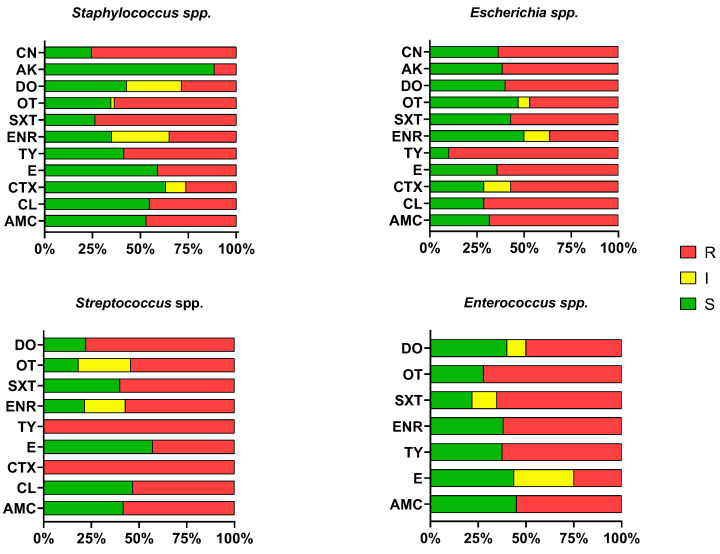
Genus-specific susceptibility (%). R-resistant; S-susceptible; I-intermediate; AMC-amoxicillin/clavulanic acid; LEX-cephalexin; CTX-cefotaxime; ERY-erythromycin; TYL-tylosin; ENR-enrofloxacin; SXT-trimethoprim/sulphamethoxazole; OXY-oxytetracycline; DOX-doxycycline; AMK-amikacin; GEN-gentamicin.

At the genus level, *Staphylococcus* spp. demonstrated high susceptibility to amikacin (88.5%) and cefotaxime (63.2%), but showed significant resistance to gentamicin (75.6%) and oxytetracycline (63.6%). In contrast, *Escherichia* spp. isolates exhibited high resistance to cephalexin (71.4%), with moderate susceptibility maintained only for enrofloxacin (50%) and doxycycline (40%). *Streptococcus* spp. exhibited a total lack of susceptibility to tylosin (100%) and cefotaxime (100%), though 57.1% of isolates remained susceptible to erythromycin. Finally, *Enterococcus* spp. showed moderate susceptibility to amoxicillin/clavulanic acid (45%) and erythromycin (43.7%), but faced high resistance levels against tylosin (62.5%) and oxytetracycline (72.2%). The data can be observed in Figure 3.

**Figure 4:**
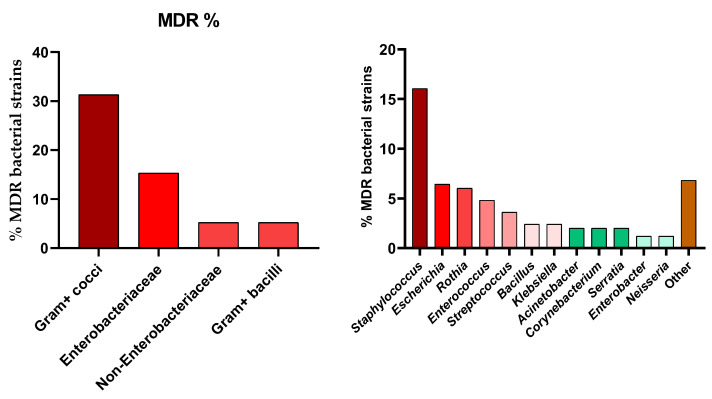
Distribution (%) of MDR isolates on bacterial groups and genera.

As demonstrated in Figure 4, from the total of 249 isolates, 57% (*n* = 142) were identified as multidrug-resistant (MDR), defined as acquired non-susceptibility to at least one agent in three or more antimicrobial categories according to the criteria established by Magiorakos et al. [28], while the remaining 43% (*n* = 107) were classified as non-MDR strains. To ensure a rigorous classification, intrinsic resistance was excluded from these calculations. The overall prevalence of multidrug resistance was disproportionately concentrated within certain taxonomic groups, with Gram-positive cocci contributing the largest share to the total MDR burden at 31.3%. This was followed by the *Enterobacteriaceae* family, which accounted for 15.3% of all MDR strains, while Non-*Enterobacteriaceae* and Gram-positive bacilli each represented 5.2% of the total resistance profile.

At the genus level, *Staphylococcus* was identified as the primary driver of resistance in the population, responsible for 16.06% of all recovered MDR bacterial strains. Other significant contributors included *Escherichia* (6.43%), Rothia (6.02%), and *Enterococcus* (4.82%). Genera such as *Streptococcus* (3.61%), *Bacillus* (2.41%), and *Klebsiella* (2.41%) showed moderate contributions. In contrast, the lowest individual contributions to the MDR total were observed in *Enterobacter* (1.2%) and *Neisseria* (1.2%), while the remaining diverse genera categorized as “Other” collectively represented 6.83% of the MDR isolates.

**Figure 5:**
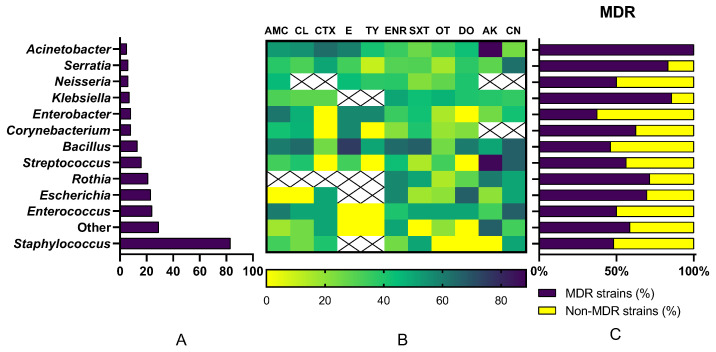
Integrated analysis of bacterial prevalence and antimicrobial resistance across 249 isolates. (**A**) Frequency distribution of recovered genera. (**B**) Heatmap of antimicrobial susceptibility (%) across 12 agents; ‘X’ denotes intrinsic resistance. (**C**) Proportion of multi-drug resistant (MDR) vs. non-MDR strains within each taxonomic group.

Figure 5 provides a comprehensive overview of the microbial landscape and the associated antimicrobial resistance burden within the study. The multifaceted analysis reveals a direct correlation between bacterial prevalence and the distribution of multidrug resistance (MDR) across 249 isolates. (A) Taxonomic distribution was dominated by *Staphylococcus* (*n* = 83; 33.33%), which also represented the largest absolute number of MDR strains (*n* = 40) recovered in the study. (B) The antimicrobial susceptibility heatmap highlights critical resistance “hotspots,” particularly against tetracyclines, where susceptibility levels were consistently low across multiple genera. The analysis also clearly delineates instances of intrinsic resistance (marked ‘X’) that define the therapeutic limits for specific genera like *Enterobacter*, *Streptococcus*, and *Enterococcus*. (C) When examining the proportion of resistance within each group, several genera with lower overall prevalence exhibited disproportionately high levels of multidrug resistance. Notably, *Acinetobacter* (100%), *Serratia* (83.33%) and *Rothia* (71.43%) showed the highest intra-genus MDR percentages, surpassing the more frequent *Staphylococcus* (48.19%). This integrated view underscores that while common pathogens drive the total volume of resistance, less frequent genera may pose a more significant challenge per isolate in a clinical setting.

## 3. Discussion

The present study demonstrated that digestive disorders were the predominant clinical presentation in pet birds, accounting for 62.1% of cases, while respiratory diseases represented 37.9%, confirming that the gastrointestinal and respiratory systems remain the primary clinical targets in ornamental avian medicine. This distribution is consistent with the high susceptibility of these systems to environmental stressors, dietary imbalances, and microbial dysbiosis in captive conditions. Notably, the lack of significant associations between pathology and host-related variables (sex, age, or season; *p* > 0.05) suggests that other factors, potentially including husbandry practices, hygiene, or varying levels of pathogen exposure, warrant further investigation as primary drivers of disease, as intrinsic biological predispositions were not evident in this cohort. Collectively, these findings suggest that clinical manifestations in pet birds are multifactorial, necessitating a focus on management-related interventions over demographic risk assessment.

A high diversity of bacterial species was recovered, with Gram-positive isolates, primarily *Staphylococcus* spp. and *Enterococcus* spp., overshadowing Gram-negative counterparts like *Escherichia coli*. This distribution is consistent with previous studies reporting that Gram-positive bacteria constitute a substantial proportion of the avian microbiota and may act as opportunistic pathogens under conditions of stress, poor hygiene, or immunosuppression [6]. The relatively high prevalence of *Staphylococcus* spp. observed in pet birds has been previously documented, with rates exceeding 20% in some populations, highlighting their ecological persistence and clinical relevance in captive avian species [6]. In parallel, the presence of Gram-negative bacteria such as *Escherichia coli* supports the concept that enteric microorganisms play a central role in avian disease, particularly in birds maintained under intensive or suboptimal management conditions [8].

Among the recovered isolates, *Escherichia coli* and *Staphylococcus aureus* emerged as pathogens of critical clinical and zoonotic significance. In this cohort, the recovery of *Escherichia coli* (9.24%) aligns with its established role as a primary driver of avian colibacillosis, particularly in cases involving enteric and systemic distress [4,29]. Previous investigations have reported high detection rates of *Escherichia coli* in pet birds, reaching up to 48.7% in cloacal samples, further supporting its epidemiological relevance in captive avian populations [8]. Similarly, *Staphylococcus aureus* has been identified as a common colonizer of avian skin and mucosa, capable of causing a wide range of infections and acting as a reservoir for zoonotic transmission, particularly in environments with close human–animal contact [6].

A notable finding of the present study was the high proportion of polymicrobial infections, suggesting that avian diseases are frequently associated with complex microbial consortia rather than isolated, single-pathogen etiologies. This observation aligns with the current understanding that disruption of the normal microbiota, often driven by stress, dietary imbalance, or inappropriate antimicrobial use, can lead to dysbiosis and subsequent overgrowth of opportunistic bacteria. In pet birds, both commensal and pathogenic microorganisms have been shown to acquire and exchange resistance traits, further complicating infection dynamics and therapeutic outcomes [8]. Such polymicrobial patterns, also described in other companion and exotic species, are typically associated with chronic disease states, increased virulence, and reduced treatment efficacy. Given the high levels of multidrug resistance (57.03%) observed in our cohort, these mixed infections highlight the clinical danger of empirical therapy and emphasize the critical necessity for comprehensive microbiological diagnostics and susceptibility-guided treatment.

The antimicrobial susceptibility results revealed a concerning pattern of resistance to commonly used antibiotics, supporting the growing evidence that pet birds act as domestic reservoirs of antimicrobial-resistant bacteria. Similar findings have been reported in previous studies, where *Escherichia coli* isolates from companion birds exhibited multidrug resistance, in some cases showing resistance to all tested antimicrobial classes [4,18]. In this study, resistance trends were primarily interpreted for the most prevalent genera to ensure statistical relevance. While certain infrequent genera (e.g., *Dermacoccus*, *Kocuria*, *Lactococcus*) showed high phenotypic resistance, these findings were interpreted with caution due to the small denominators (*n* < 5). Such results are presented as individual clinical findings rather than representative resistance ‘hotspots’ or broader epidemiological trends. While the current study focused on phenotypic resistance, previous investigations have identified extended-spectrum β-lactamase (ESBL)-producing *Escherichia coli* in pet birds, with all confirmed isolates demonstrating multidrug resistance and carrying resistance genes such as *bla*_TEM_ and *bla*_CTX-M_, highlighting the genetic basis and dissemination potential of resistance traits [8]. In parallel, Gram-positive pathogens such as *Staphylococcus aureus* have shown resistance to critical antimicrobials, including methicillin and vancomycin, further emphasizing the clinical and public health implications of resistant bacteria circulating in avian hosts [6,30]. These resistance patterns are likely driven by the widespread and often unregulated use of antimicrobials in avian practice, including prophylactic administration, empirical treatment, and subtherapeutic dosing, which collectively create selective pressure favoring the emergence and persistence of multidrug-resistant strains.

The high resistance observed against oxytetracycline (68.4%) widespread non-susceptibility to macrolides (e.g., 61.2% in Gram-positive cocci) in the present study is consistent with the frequent use of these antimicrobial classes in ornamental birds. In avian practice, both macrolides and tetracyclines are commonly administered as over-the-counter water-soluble formulations, frequently without veterinary supervision. This mode of administration often results in sub-therapeutic dosing and prolonged exposure, creating optimal conditions for the selection and persistence of resistant bacterial populations. Such selective pressure is particularly evident among Gram-positive organisms, including *Staphylococcus* spp. and *Enterococcus* spp., which represented a substantial proportion of isolates in the current study. These findings align with previous observations that inappropriate antimicrobial use in companion birds significantly contributes to the emergence of resistance within commensal and opportunistic microbiota [31].

The increasing prevalence of antimicrobial resistance observed in this study also highlights the urgent need to explore alternative therapeutic strategies in avian medicine. In this context, plant-derived compounds have gained attention due to their antimicrobial properties, with several studies demonstrating that essential vegetal extracts can exhibit inhibitory effects against *Staphylococcus aureus*, in some cases comparable to conventional antibiotics [32].

The findings of this study reinforce the role of pet birds as potential reservoirs of antimicrobial-resistant bacteria within a One Health framework, emphasizing the interconnectedness of animal, human, and environmental health. Close contact between companion birds and their owners facilitates the transmission of opportunistic and resistant pathogens through direct handling, aerosols, or contaminated surfaces, thereby increasing the risk of zoonotic dissemination [33,34,35]. In addition to household transmission, veterinary environments have also been identified as important reservoirs and dissemination points for resistant bacteria such as the multidrug-resistant *Enterobacteriaceae* identified in this cohort, highlighting the potential for cross-species and environmental spread of antimicrobial resistance in clinical settings. These findings highlight the need for integrated surveillance strategies and infection control measures across veterinary and human healthcare systems to mitigate the spread of resistant pathogens.

From a clinical perspective, the high prevalence of bacterial infections and the observed antimicrobial resistance patterns highlight the necessity of implementing targeted diagnostic and therapeutic approaches in avian practice. Empirical antimicrobial therapy, particularly with commonly used agents such as tetracyclines and macrolides, may be ineffective and contribute to further resistance development, especially in the presence of multidrug-resistant strains [8]. Therefore, routine bacteriological culture and antimicrobial susceptibility testing should be strongly encouraged prior to treatment initiation, in order to optimize therapeutic outcomes and reduce inappropriate antimicrobial use. Additionally, improving husbandry practices, hygiene conditions, and owner education may play a critical role in preventing disease occurrence and limiting the spread of resistant pathogens in companion bird populations.

The identification of several atypical bacterial taxa in this study highlights a shifting landscape of avian and zoonotic risk. Notably, *Gallibacterium anatis* has transitioned from a commensal of the poultry respiratory and reproductive tracts to a recognized pathogen in both commercial and ornamental birds, with rare documented cases of human infection indicating its zoonotic potential [36]. Similarly, *Lactococcus garvieae*, originally established as a major global threat in the aquaculture industry, is increasingly identified in subclinical inflammations across diverse hosts, including poultry, swine, and domestic carnivores. These animal reservoirs serve as critical sources for human transmission, where *Lactococcus garvieae* is now recognized as a significant opportunistic pathogen associated with high morbidity and mortality [37,38]. Furthermore, *Leclercia adecarboxylata* has emerged as a ‘novel’ rare human pathogen; while traditionally isolated in polymicrobial infections among immunocompetent patients, it is increasingly implicated in severe clinical syndromes, including septicemia, peritonitis, and urinary tract infections, particularly within immunocompromised populations [39,40,41].

The identification of specific bacterial lineages in this study highlights a significant public health risk, as pet birds serve as critical reservoirs for pathogens capable of crossing the species barrier. Avian Pathogenic *Escherichia coli* (APEC) and Shiga toxin-producing *Escherichia coli* (STEC) represent a primary concern, as these strains can be transferred to humans, potentially leading to severe conditions such as hemolytic uremic syndrome (HUS), urinary tract infections (UTIs), and diarrhea [3]. Furthermore, *Klebsiella pneumoniae* emerged as a pathogen of high clinical relevance; while it may be isolated from the feces of seemingly healthy *Passeriformes* and *Psittaciformes*, often manifesting as respiratory infections in the birds, it is a major cause of nosocomial meningitis, pneumonia, and soft tissue infections in humans [3]. The presence of *Staphylococcus aureus* and methicillin-resistant *Staphylococcus aureus* (MRSA) further compounds this risk. Primarily colonizing the cloacae and nostrils of various avian species, these organisms not only act as pathogenic agents within the birds themselves but also pose a direct danger to human contacts, who may contract life-threatening infections such as MRSA-induced endocarditis [42,43]. While molecular typing for STEC or MRSA was not performed in this study, the isolation of *Escherichia coli* and *Staphylococcus aureus* from clinical cases highlights the potential for these birds to carry lineages of public health concern, as documented in similar avian populations. The presence of *Enterococcus* spp. in this study further complicates the clinical landscape due to their environmental resilience and capacity for genetic exchange. Free-ranging and captive birds act as significant reservoirs for vancomycin- and gentamicin-resistant isolates, serving as zoonotic sources of multidrug resistance [44]. Specifically, *Enterococcus faecalis* is a formidable nosocomial pathogen that can be silently carried by ornamental birds, facilitating the dissemination of virulence and resistance genes to humans [45]. This colonization poses a substantial risk for human endocarditis and urinary tract infections, particularly when strains exhibit high-level aminoglycoside resistance. Such findings underscore a critical ‘One Health’ challenge, where avian hosts facilitate the persistence of difficult-to-treat infections in human contacts [44,45].

Taken together, these findings reinforce the broader role of companion birds within the antimicrobial resistance landscape. Overall, these findings indicate that pet birds represent a relevant component of the antimicrobial resistance landscape. While direct transmission was not evaluated in this study, the presence of resistant taxa suggests they may serve as potential reservoirs within the One Health network.

Despite the valuable insights provided by this study, several limitations should be acknowledged. The investigation was conducted within a single geographic region, which may limit the generalizability of the findings to broader avian populations. Additionally, molecular characterization of resistance mechanisms was not performed, restricting the ability to identify specific resistance genes and transmission pathways, which are increasingly recognized as critical components in understanding antimicrobial resistance dynamics in both clinical and environmental contexts [46]. Furthermore, standardized antimicrobial susceptibility breakpoints for exotic avian species remain limited, potentially affecting the interpretation of resistance profiles and comparability with other studies. Future research integrating molecular approaches and standardized methodologies is needed to better elucidate the epidemiology and clinical significance of antimicrobial resistance in pet birds.

## 4. Materials and Methods

### 4.1. Animals and Study Design

The present study was conducted over a three-year period, from November 2022 to March 2026, and included a total of 198 pet birds (the primary analytical unit) referred to the Exotic Animal Clinic at the Faculty of Veterinary Medicine in Cluj-Napoca, Romania. Each patient underwent a thorough clinical examination performed by specialized medical staff. Based on the clinical findings, samples were collected and submitted to the Department of Microbiology of the same faculty for bacteriological analysis.

Specimens were obtained using sterile cotton swabs with Amies transport medium (DeltaLab, Barcelona, Spain) from birds presenting with digestive and respiratory disorders. Each sample was accompanied by a detailed dispatch note containing relevant information regarding the owner and the animal (species, age, and sex), the season of presentation, the type of specimen collected, and whether antimicrobial therapy had been administered prior to bacteriological testing. To ensure diagnostic integrity, every specimen was appropriately labeled, transported, and processed on the same day of collection. A clinically relevant bacterial infection was defined as the isolation of a primary pathogen or the predominant growth of opportunistic bacteria in the presence of matching clinical signs. Isolates considered to be environmental contaminants or part of the normal commensal flora without evidence of overgrowth were excluded from the final resistance analysis.

### 4.2. Microbial Identification

Upon arrival at the laboratory, all specimens were registered and underwent immediate microbiological processing. The samples were inoculated using the streak plate method onto both general and selective culture media for pathogen isolation, including Columbia agar supplemented with 10% sheep blood (BioMaxima S.A., Lublin, Poland), UriSelect medium (Bio-Rad Laboratories Inc., Hercules, CA, USA), and MacConkey agar (Merck, Darmstadt, Germany). The plates were incubated aerobically at 37 °C for 24–48 h. After incubation, colonies were evaluated based on their cultural characteristics, followed by microscopic examination using Gram staining.

Preliminary bacterial classification was performed using the 3% potassium hydroxide (KOH) test, the slide catalase test with 3% hydrogen peroxide for Gram-positive cocci, and the oxidase test (Rotitest^®^ Oxidase strips, Carl Roth, Karlsruhe, Germany) for Gram-negative rods. In cases where no bacterial growth was observed after 48 h of incubation, the samples were considered negative.

For definitive identification, 24 h pure cultures were analyzed using the MALDI Biotyper^®^ Sirius System (Bruker, Ettlingen, Germany). This proteomic approach utilizes mass spectrometry to identify bacterial strains by comparing the unique protein spectra of the analyzed isolates against an extensive database of reference spectra. A log(score) ≥ 2.0 was considered a definitive species-level identification. For isolates yielding scores between 1.7 and 2.0, the identification process was performed in duplicate to ensure consistency. Furthermore, to safeguard against ambiguous results, every isolate was verified through Gram staining and evaluation of cultural characteristics to ensure phenotypic agreement with the proteomic identification. All procedures were performed in strict accordance with the manufacturer’s guidelines. Following identification, the confirmed bacterial isolates were preserved in cryotubes containing 60% glycerol broth and stored at −20 °C for subsequent analyses. Due to the absence of ultra-low temperature freezers (−80 °C), viability was ensured through a systematic maintenance program involving monthly subculturing onto fresh media. Before any subsequent analysis or antimicrobial susceptibility testing, isolates were thawed and subcultured to verify purity and typical growth characteristics.

### 4.3. Antimicrobial Susceptibility Testing

The antimicrobial susceptibility profile for each isolated strain was determined using the Kirby–Bauer disk diffusion method, performed in accordance with EUCAST guidelines [47]. To ensure standardized results, bacterial suspensions were prepared in sterile saline (0.9% NaCl, Sigma Aldrich, Darmstadt, Germany) to achieve a turbidity of 0.5 McFarland. These suspensions were then uniformly inoculated onto Mueller–Hinton (MH) agar plates (Merck, Darmstadt, Germany) using the three-section streaking technique [47]. To establish the accuracy and reproducibility of the results, quality control was performed using the following reference strains: *Escherichia coli* ATCC 25922, *Staphylococcus aureus* ATCC 25923, and *Pseudomonas aeruginosa* ATCC 27853. All results were verified to be within the acceptable limits defined by CLSI before clinical isolate data were recorded.

A total of 11 antimicrobial agents (Liofilchem, Teramo, Italy) representing six major therapeutic classes were selected for susceptibility testing: β-lactams, including a penicillin/β-lactamase inhibitor combination (amoxicillin-clavulanic acid 20/10 μg) and cephalosporins (cefalexin 30 μg, cefotaxime 30 μg); aminoglycosides (gentamicin 10 μg, amikacin 30 μg); macrolides (erythromycin 15 μg, tylosin 30 μg); fluoroquinolones (enrofloxacin 5 μg); tetracyclines (oxytetracycline 30 μg, doxycycline 30 μg); and folate pathway inhibitors (trimethoprim-sulfamethoxazole 1.25/23.75 μg). The antibiotic disks were positioned radially on the inoculated plates.

Plates were incubated at 35 ± 1 °C for 16–20 h, after which inhibition zone diameters were measured. Isolates were interpreted as susceptible (S), intermediate (I), or resistant (R) according to EUCAST [27] and CLSI VET01S ED7:2024 [48] clinical standards.

### 4.4. Statistical Analysis

Data were organized using Microsoft Excel 2021 (Microsoft Corporation, Redmond, WA, USA) and subjected to statistical analysis with Epi Info™ 7.2 (CDC, Atlanta, GA, USA) and GraphPad Prism 8 (GraphPad Software, San Diego, CA, USA). Furthermore, all the values were analyzed using descriptive statistics to determine the frequency and percentage distribution of all variables. To assess the associations between clinical pathologies and demographic factors (sex, age, and season), Chi-square (χ^2^) tests of independence were performed. In cases where expected cell frequencies were less than 5, Fisher’s Exact Test was applied to ensure analytical validity. All tests were two-tailed with a significance threshold (alpha) set at *p* < 0.05. Exploratory associations between clinical presentations and demographic variables were evaluated using Odds Ratios (OR) with 95% Confidence Intervals (CI).

## 5. Conclusions

This study suggests that clinically affected pet birds may serve as significant hosts for diverse bacterial populations, including both Gram-positive and Gram-negative species with notable clinical and zoonotic potential. The predominance of digestive disorders, together with the high frequency of opportunistic pathogens such as *Escherichia coli* and *Staphylococcus aureus*, highlights the critical role of microbial imbalances and environmental factors in the pathogenesis of disease in captive avian species.

The detection of substantial antimicrobial resistance, including multidrug-resistant strains, raises serious concerns regarding therapeutic efficacy and public health, particularly in the context of close human–animal interactions. These findings reinforce the importance of adopting a One Health approach, integrating veterinary, environmental, and human health perspectives to better understand and control the spread of resistant bacteria.

Routine microbiological diagnostics, including culture and antimicrobial susceptibility testing, should be prioritized in avian clinical practice to guide targeted therapy and reduce inappropriate antimicrobial use. In parallel, improved husbandry practices, enhanced biosecurity measures, and increased awareness among bird owners are essential to minimize disease occurrence and transmission.

Future research should focus on molecular characterization of resistance mechanisms and the development of standardized guidelines for antimicrobial use in exotic species to mitigate the emergence and dissemination of antimicrobial resistance in pet bird populations.

## Figures and Tables

**Table 1:** Data concerning the population studied based on gender, age, season, pathology, and previous antibiotic exposure.

Birds %					Total
Gender	Male 59 (*n* = 117)		Female 41 (*n* = 81)		100 (*n* = 198)
Age	Juvenile 30.3 (*n* = 60)		Adult 69.7 (*n* = 138)		100 (*n* = 198)
Season	Spring 22.7 (*n* = 45)	Summer 27.3 (*n* = 54)	Autumn 23.7 (*n* = 47)	Winter 26.3 (*n* = 52)	100 (*n* = 198)
Pathology	Digestive 62.1 (*n* = 123)		Respiratory 37.9 (*n* = 75)		100 (*n* = 198)
Antibiotic usage	Yes 21.7 (*n* = 43)		No 78.3 (*n* = 155)		100 (*n* = 198)

**Table 2:** Distribution of pet birds’ pathologies according to age, gender, and seasonal variations.

		Digestive	Respiratory	Total
Gender (%)	Male	59.3 (*n* = 73)	58.7 (*n* = 44)	100 (*n* = 198)
	Female	40.7 (*n* = 50)	41.3 (*n* = 31)	
Age (%)	Young	31.7 (*n* = 39)	28 (*n* = 21)	100 (*n* = 198)
	Adult	68.3 (*n* = 84)	72 (*n* = 54)	
Season (%)	Spring	24.4 (*n* = 30)	20 (*n* = 15)	100 (*n* = 198)
	Summer	30 (*n* = 37)	22.7 (*n* = 17)	
	Autumn	23.6 (*n* = 29)	24 (*n* = 18)	
	Winter	22 (*n* = 27)	33.3 (*n* = 25)	
Total (*n*)		62.1 (*n* = 123)	37.9 (*n* = 75)	100 (*n* = 198)

**Table 3:** Bacterial strains isolated from pet birds distributed across pathologies.

Group	Genus	Species	Digestive (*n*)	Respiratory (*n*)	Total (*n*)
Gram + cocci	*Dermacoccus*	*Dermacoccus nishinomiyaensis*	-	3	3
	*Enterococcus*	*Enterococcus faecalis*	18	-	18
		*Enterococcus faecium*	4	2	6
	*Kocuria*	*Kocuria kristinae*	-	2	2
		*Kocuria rhizophila*	-	1	1
		*Kocuria rosea*	-	1	1
	*Lactococcus*	*Lactococcus garvieae*	-	1	1
	*Micrococcus*	*Micrococcus luteus*	-	3	3
	*Rothia*	*Rothia dentocariosa*	1	2	3
		*Rothia kristinae*	5	1	6
		*Rothia nasimurium*	3	9	12
	*Staphylococcus*	*Staphylococcus aureus*	1	5	6
		*Staphylococcus borealis*	-	3	3
		*Staphylococcus delphini*	2	-	2
		*Staphylococcus epidermidis*	1	3	4
		*Staphylococcus equorum*	1	-	1
		*Staphylococcus gallinarum*	1	3	4
		*Staphylococcus haemolyticus*	6	20	26
		*Staphylococcus hominis*	-	1	1
		*Staphylococcus hyicus*	-	1	1
		*Staphylococcus intermedius*	-	1	1
		*Staphylococcus kloosii*	1	-	1
		*Staphylococcus saprophyticus*	2	-	2
		*Staphylococcus sciuri*	6	5	11
		*Staphylococcus simulans*	-	1	1
		*Staphylococcus vitulinus*	2	-	2
		*Staphylococcus warneri*	9	3	12
		*Staphylococcus xylosus*	5	-	5
	*Streptococcus*	*Streptococcus gallolyticus*	-	2	2
		*Streptococcus mitis*	-	4	4
		*Streptococcus pluranimalium*	1	-	1
		*Streptococcus salivarius*	5	1	6
		*Streptococcus sanguinus*	1	-	1
		*Streptococcus suis*	1	1	2
G + bacilli	*Bacillus*	*Bacillus cereus*	3	-	3
		*Bacillus circulans*	1	-	1
		*Bacillus licheniformis*	-	1	1
		*Bacillus pumilus*	1	-	1
		*Bacillus* spp.	4	2	6
		*Bacillus velezensis*	1	-	1
	*Corynebacterium*	*Corynebacterium amycolatum*	1	-	1
		*Corynebacterium durum*	-	1	1
		*Corynebacterium falsenii*	-	1	1
		*Corynebacterium* spp.	3	2	5
	*Lactobacillus*	*Lactobacillus crispatus*	1	-	1
		*Lactobacillus johnsonii*	1	-	1
		*Lactobacillus salivarius*	1	-	1
	*Microbacterium*	*Microbacterium aerolatum*	1	-	1
Enterobacteriaceae	*Enterobacter*	*Enterobacter asburiae*	-	1	1
		*Enterobacter cloacae*	3	-	3
		*Enterobacter hormaechei*	1	2	3
		*Enterobacter kobei*	1	-	1
	*Escherichia*	*Escherichia coli*	22	1	23
	*Klebsiella*	*Klebsiella oxytoca*	1	1	2
		*Klebsiella pneumoniae*	3	2	5
	*Kosakonia*	*Kosakonia cowanii*	1	-	1
	*Leclercia*	*Leclercia adecarboxylata*	1	-	1
	*Mixta*	*Mixta gaviniae*	1	-	1
	*Proteus*	*Proteus mirabilis*	-	3	3
		*Proteus vulgaris*	1	-	1
	*Raoultella*	*Raoultella terrigena*	1	-	1
	*Serratia*	*Serratia marcescens*	3	3	6
Non-Enterobacteriaceae	*Acinetobacter*	*Acinetobacter baumannii*	1	-	1
		*Acinetobacter pittii*	1	1	2
		*Acinetobacter seifertii*	1	-	1
		*Acinetobacter ursingii*	-	1	1
	*Aeromonas*	*Aeromonas caviae*	1	-	1
		*Aeromonas salmonicida*	1	1	2
	*Gallibacterium*	*Gallibacterium anatis*	2	-	2
	*Neisseria*	*Neisseria denitrificans*	-	1	1
		*Neisseria sicca*	-	1	1
	*Pseudomonas*	*Pseudomonas monteilii*	1	-	1
Total			141	108	249

## Data Availability

All data generated or analysed during this study are included in this published article.
